# Oridonin inhibits DNMT3A R882 mutation-driven clonal hematopoiesis and leukemia by inducing apoptosis and necroptosis

**DOI:** 10.1038/s41420-021-00697-5

**Published:** 2021-10-18

**Authors:** Min Liao, Qiongye Dong, Ruiqing Chen, Liqian Xu, Yuxuan Jiang, Zhenxing Guo, Min Xiao, Wei He, Changcai Cao, Ronghua Hu, Wanling Sun, Hong Jiang, Jianwei Wang

**Affiliations:** 1 School of Pharmaceutical Sciences, Tsinghua University, 100084 Beijing, China; 2 Key Laboratory of Intelligent Information Processing, Advanced Computer Research Center, Institute of Computing Technology, Chinese Academy of Sciences, 100190 Beijing, China; 3 Department of Hematology/Oncology, First Hospital of Tsinghua University, 100016 Beijing, China; 4 Department of Hematology, Tongji Hospital, Tongji Medical College, Huazhong University of Science and Technology, 430030 Wuhan, China; 5 Shandong Hongmai Biotechnology Co., Ltd. Room 1201, building B, Research Institute of Tianjin University, No. 51, Lutai Avenue, Zibo High tech Zone, 255000 Tianjin, China; 6 Department of Hematology, Xuanwu Hospital, Capital Medical University, 100053 Beijing, China; 7 Kidney Disease Center, the First Affiliated Hospital, College of Medicine, Zhejiang University, 310003 Hangzhou, China

**Keywords:** Acute myeloid leukaemia, High-throughput screening

## Abstract

DNA (cytosine-5)-methyltransferase 3A (DNMT3A) mutations occur in ~20% of de novo acute myeloid leukemia (AML) patients, and >50% of these mutations in AML samples are heterozygous missense alterations within the methyltransferase domain at residue R882. DNMT3A R882 mutations in AML patients promote resistance to anthracycline chemotherapy and drive relapse. In this study, we performed high-throughput screening and identified that oridonin, an ent-kaurene diterpenoid extracted from the Chinese herb *Rabdosia rubescens*, inhibits DNMT3A R882 mutant leukemic cells at a low-micromolar concentration (IC_50_ = 2.1 µM) by activating both RIPK1-Caspase-8-Caspase-3-mediated apoptosis and RIPK1-RIPK3-MLKL-mediated necroptosis. The inhibitory effect of oridonin against DNMT3A R882 mutant leukemia cells can also be observed in vivo. Furthermore, oridonin inhibits clonal hematopoiesis of hematopoietic stem cells (HSCs) with Dnmt3a R878H mutation comparing to normal HSCs by inducing apoptosis and necroptosis. Overall, oridonin is a potential and promising drug candidate or lead compound targeting DNMT3A R882 mutation-driven clonal hematopoiesis and leukemia.

## Introduction

DNA (cytosine-5)-methyltransferase 3A (DNMT3A) is one of the most frequently mutated genes in age-related clonal hematopoiesis (ARCH) which is related to the increases in the risk of hematological malignancies and the all-cause mortality [1–3]. DNMT3A mutations are also detected in ~20% of acute myeloid leukemia (AML) patients, and more than half of these mutations occur at the arginine 882 which is commonly mutated to histidine (R882H) or cysteine (R882C) [4]. AML patients with DNMT3A R882 mutation have an adverse outcome by promoting resistance to anthracycline chemotherapy via impaired nucleosome remodeling [5], and the frequency of DNMT3A mutant hematopoietic stem cells (HSCs) in AML patients increases gradually from diagnosis to relapse [6], suggesting that DNMT3A mutant cells survive chemotherapy and drive relapse. Thus, pharmacologic inhibition or elimination of DNMT3A mutant cells may be an effective way to eradicate the DNMT3A mutant AML.

DNMT3A is one of the de novo DNA methyltransferases, and DNMT3A R882H AML cells exhibit decreased de novo methyltransferase activity and markedly hypomethylation throughout the genomes of these cells at specific CpGs [7]. While the global 5-methylcytosine (5mC) levels show no difference in the whole genome between control and Dnmt3a^−/−^ HSCs, and Dnmt3a loss in HSCs leads to both hyper- and hypomethylation [8]. However, it seems to be contradictory that hypomethylating agents, including azacytidine and decitabine, exhibit therapeutic effect on DNMT3A mutant AML patients [9, 10], which may be due to the changed flanking sequence preference of the aberrant DNMT3A enzyme that results in abnormal hypermethylation at some specific gene loci contributing to leukemogenesis [11, 12]. In addition, high-dose anthracycline therapies show certain efficacy for DNMT3A mutant leukemia in AML clinical trials, while the increased toxicity limits its application [13].

Another strategy is to inhibit aberrantly activated genes or the related signaling pathways caused by DNMT3A mutations. The histone methyltransferase DOT1L and homeobox cluster A and B genes were found to be upregulated in DNMT3A mutant AML cells [14, 15]. Pharmacologic inhibition of DOT1L by small molecules can restore the expression of HOXA and HOXB genes, which proves that DOT1L inhibitors are potential drug candidates for targeting DNMT3A mutant AML [14, 16]. Furthermore, the mTOR pathway inhibition induced by rapamycin also inhibits the proliferation of mouse Dnmt3a R878H cells and human DNMT3A mutant AML cells [17]. Despite the above-mentioned research, more efficient therapies or new target therapies are still worthy of further research.

In this study, we performed high-throughput screening from FDA-approved drug libraries and natural product libraries to obtain inhibitors targeting DNMT3A R882 mutant leukemia cells. Finally, we identified oridonin, an ent-kaurene diterpenoid extracted from the Chinese herb *Rabdosia rubescens* [18], which inhibits DNMT3A R882 mutant leukemic cells at a low-micromolar concentration (IC_50_ = 2.1 µM) by activating both RIPK1-Caspase-8-Caspase-3-mediated apoptosis and RIPK1-RIPK3-MLKL-mediated necroptosis. The inhibitory effect of oridonin against DNMT3A R882 mutant cells and Dnmt3a R878H cells can also be observed in a xenograft model and transplantation assay mimicking Dnmt3a R878H-based clonal hematopoiesis, respectively. Overall, oridonin is a potential and promising drug candidate or lead compound targeting DNMT3A R882 mutation-driven clonal hematopoiesis and leukemia.

## Results

### Construction of reporter cell lines for screening

**Fig. 1:**
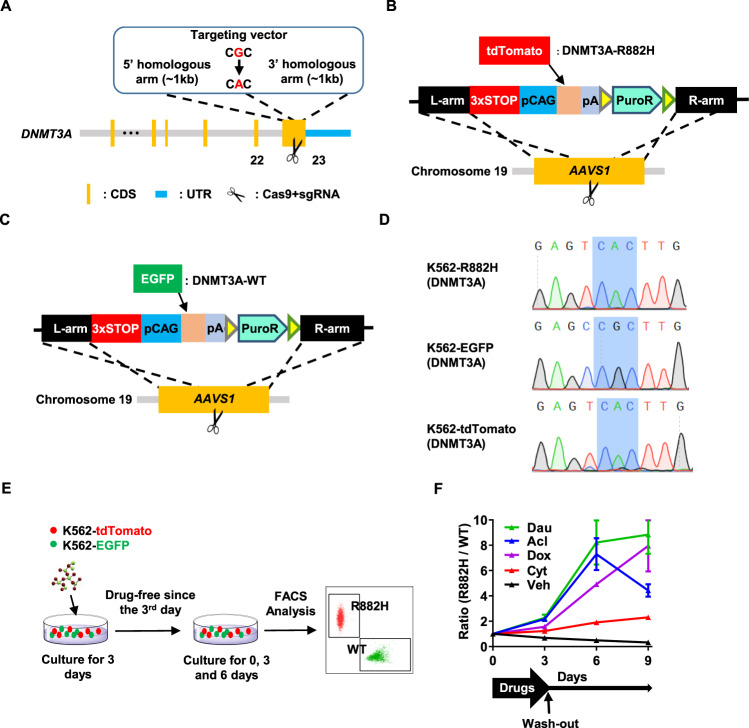
Construction of reporter cell lines for screening. **A** The schematic diagram depicts the targeting strategy to generate the K562 cell line with *DNMT3A* R882H mutation (K562-R882H). **B** The schematic diagram shows that a tdTomato reporter gene was inserted into adeno-associated virus integration site 1(*AAVS1*) allele of K562-R882H cell line to generate K562-tdTomato (R882H) cell lines. **C** The schematic diagram displays that an EGFP reporter gene was inserted into adeno-associated virus integration site 1(*AAVS1*) allele of K562-WT cell line to generate K562-EGFP (WT) cell lines. **D** Sequencing analysis of the genomic site encoding *DNMT3A* R882 (the shadow) in three cell lines developed in this study (K562-R882H, K562-EGFP, and K562-tdTomato). **E**, **F** K562-EGFP and K562-tdTomato cells at a ratio of 1:1 were seeded into 96-well plates at a density of 1×10^4^ cells per well. Cells were treated with commonly used chemotherapy drugs, including Daunorubicin (Dau, 5 µM), Aclarubicin (Acl, 5 µM), Doxorubicin (Dox, 5 µM), or Cytarabine (Cyt, 0.2 µM), and vehicle (Veh) for 3 days, respectively. Three days later, a third of the cultures were analyzed by flow cytometry. The drugs and vehicle in the remaining cultures were removed and replaced by a fresh culture medium. Then these cells were cultured for another 3 days or 6 days before being detected by flow cytometry. **E** Experimental design. **F** The line plots depict the ratio of R882H cells to WT cells (the vertical axis) with the indicated treatment that changed over time.

Given that the human *DNMT3A* R882H (mouse R878H) is a driver mutation for clonal hematopoiesis and one of the major risk factors for myeloid leukemia progression [1–3, 6], it is of great importance to obtain small molecule(s) inhibiting hematopoietic cells carrying *DNMT3A* R882H. To screen such compound(s), we generated a K562 cell line with *DNMT3A* R882H mutation (hereafter named K562-R882H or R882H), wherein guanine at the 2645 site in the exon 23 of DNMT3A was replaced by an adenine to encode R882H mutation using CRISPR-Cas9 system (Fig. 1A). Then, we developed two reporter cell lines: K562-tdTomato, wherein a red fluorescent protein (tdTomato) was inserted into *the AAVS1* gene site of K562-R882H (Fig. 1B), and K562-EGFP, wherein an enhanced green fluorescent protein (EGFP) was inserted into *the AAVS1* gene site of the WT K562 cell line (Fig. 1C). The *DNMT3A* R882H mutation in K562-R882H and K562-tdTomato cells was confirmed by Sanger sequencing, and the result showed that the 2645 site in the exon 23 of DNMT3A was successfully edited by CRISPR-Cas9 in these two cell lines (Fig. 1D).

Previous studies suggested that *DNMT3A* mutations lead to chemotherapy resistance in acute myeloid leukemia [5, 6]. To evaluate the response of DNMT3A R882H cells used in this study to chemotherapy drugs, K562-tdTomato, and K562-EGFP cells were mixed at the ratio of 1:1 and the mixed cells were treated with different commonly used chemotherapy drugs for the indicated time (Fig. 1E). The result displayed that the frequency of R882H cells exhibits a downward trend over time without chemotherapy stress. In contrast, the percentage of R882H cells increases when the cultures were treated with daunorubicin, aclarubicin, doxorubicin, or cytarabine, indicating that R882H cells obtain a survival advantage over WT cells under chemotherapy stress (Fig. 1F). This result is consistent with the previous reports that *DNMT3A* R882H mutation results in chemotherapy resistance [5, 6], which proves that this cell line model works well. Thus, this model can be utilized to screen small molecules targeting DNMT3A R882H cells.

### High-throughput screening of small molecules that inhibit cells carrying DNMT3A R882H mutation

Drug repurposing, namely new uses for old drugs, has been encouraged in recent years for the intensively investigated targets, safety, and pharmacokinetics of the approved drugs [19]. In addition, early phase clinical trials are permitted to be bypassed for the routine clinical used drug for other purposes, which save both time and money [20]. Therefore, in this study, we focused on screening for inhibitors targeting DNMT3A R882 mutant cells from the FDA-approved drug library and natural product library.

**Fig. 2:**
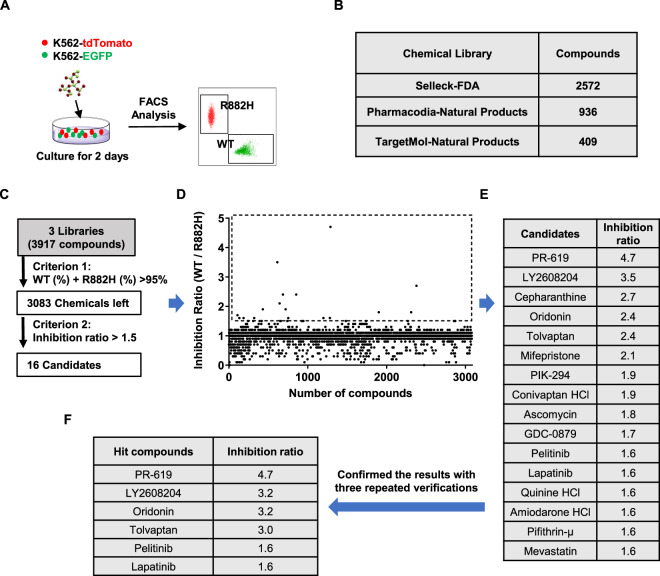
High-throughput screening of small molecules that inhibit cells carrying DNMT3A R882H mutation. **A** High-throughput screening (HTS) was performed to obtain small molecules against *DNMT3A* R882H cells. In all, 5 × 10^3^ K562-EGFP cells along with 5 × 10^3^ K562-tdTomato cells were seeded into a 96-well plate at a density of 1 × 10^4^ cells per well. Cells were treated with chemicals (5 µM) from different compound libraries. Three days later, all cultures were analyzed by flow cytometry with a high throughput sampler. **B** The table displays a total of 3917 chemicals that we screened from three chemical libraries, including an FDA-approved drug library and two natural products libraries. **C** HTS assay flowchart. The first criterion used for the screen was that the percentage of WT cells plus R878H cells should >95%. In total, 16 candidates were obtained when we applied the second criterion that the ratio of WT cells to R882H cells should be over 1.5. **D** The scatter plot shows the overall screening results and 16 candidates with an inhibition ratio of more than 1.5 are marked with a dotted frame. **E** The table displays the name and inhibition ratio value of the 16 candidates. **F** The primary screen results were further confirmed with three repeated verifications and 6 hit compounds were selected.

First of all, we mixed K562-tdTomato and K562-EGFP cells at the ratio of 1:1 and screened three libraries (one FDA-approved drug library and two natural product libraries, Table S1–3) for small molecule(s) that can inhibit K562-tdTomato cells by detecting the ratio of EGFP and tdTomato (Fig. 2A, B). A total of 3917 compounds were screened and 834 chemicals were ruled out for the possible nonspecific effect on the reporter protein when the percentage of WT cells plus R878H cells was no >95% (Fig. 2C). Then 16 candidates were achieved when we used another criterion that the ratio of WT cells to R882H cells should be over 1.5 (Fig. 2D, E). The possible false-positive candidates were excluded by verifying the results three times, and we finally obtained 6 hit compounds, including PR-619, LY2608204, oridonin, tolvaptan, pelitinib, and lapatinib (Fig. 2F). The above results suggest that the high-throughput screening assay was feasible and reliable, and this method can also be applied to screen for chemicals targeting other mutant genes.

### Oridonin induces apoptosis in DNMT3A R882H cells

**Fig. 3:**
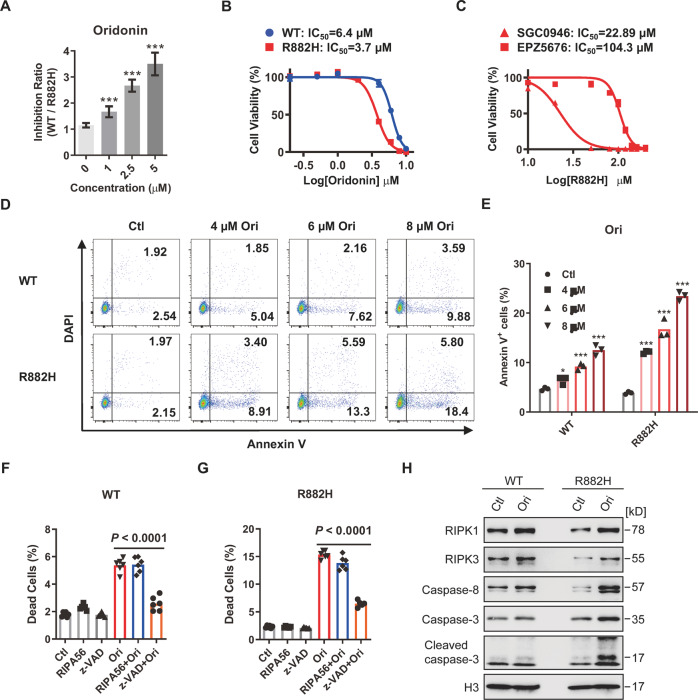
Oridonin induces apoptosis in DNMT3A R882H cells. **A** The histogram shows the inhibitory effect of oridonin against R882H cells at different concentrations. The inhibition ratio shown in the vertical axis was calculated using the percentage of WT cells divided by R882H cells. Briefly, K562-EGFP cells along with K562-tdTomato cells at a ratio of 1:1 were seeded into 96-well plates at a density of 1 × 10^4^ cells per well. Then, cells were treated in triplicate with the indicated compounds at different concentrations, and all cultures were subjected to FACS analysis 48 h later. All data above are shown as mean ± SD and compared to the vehicle control; *P < 0.05, **P < 0.01, and ***P < 0.001. **B** The inhibitory effect of oridonin against R882H cells was evaluated using a CCK-8 kit. The half-maximal inhibitory concentration (IC_50_) of oridonin against R882H cells is displayed in the line plots. Data are represented as mean ± SD, *n* = 3 per concentration from two biological replicates. **C** The inhibitory effect of SGC0946 and EPZ5676 against R882H cells was evaluated using a CCK-8 kit. The IC_50_ of two compounds against R882H cells is displayed in the line plots. Data are represented as mean ± SD, *n* = 3 per concentration from two biological replicates. **D**, **E** Oridonin (Ori) induces apoptosis in WT and R882H cells. All cells (1 × 10^4^ cells per well) were incubated with 4 µM, 6 µM, 8 µM Ori or an equal volume of vehicle. After 48 h of incubation, cells were harvested and stained with Annexin V/DAPI before being subjected to flow cytometric analysis. **D** Representative dot plots of WT and R882H cells for apoptotic analysis in Ori-treated group and control (Ctl) group. **E** The histogram displays the frequency of apoptotic cells (Annexin V^+^) in WT and R882H cells with the indicated treatment. All data above are shown as mean ± SD and compared to the vehicle control; *P < 0.05, **P < 0.01, and ***P < 0.001. **F**, **G** 1 × 10^4^ WT or R882H cells were treated with necroptosis inhibitor (RIPA56, 20 µM), apoptotic inhibitor (z-VAD, 20 µM), Ori (6 µM), RIPA56 and Ori, or z-VAD and Ori for 48 h. Then, dead cells (DAPI^+^) were evaluated by FACS after DAPI staining. The histograms show the percentage of dead cells in WT (**F**) and R882H (**G**) cells with the indicated treatment. Data are represented as mean ± SD from three independent experiments. **H** Immunoblot analysis of the proteins involved in the apoptosis pathway in WT and R882H cells after treatment with 6 µM Ori for 48 h.

Given that a good drug candidate usually exhibits a dose-dependent effect, we then examined the inhibitory effect of 6 hit compounds against R882H cells over a concentration range. The result showed that oridonin, PR-619 and tolvaptan exhibit a favorable concentration-dependent inhibitory effect from 0 to 5 µM, and the maximum inhibition ratio of these compounds is over 3 (Figs. 3A and S1A–B). While the selective inhibitory effect of pelitinib against R882H cells decreases when the concentration is over 2.5 µM (Fig. S1C), and both LY2608204 and lapatinib display no selective inhibitory effect under 5 µM (Fig. S1D–E).

To further confirm the above results, WT and R882H cells were treated with the 6 small compounds separately and measured the cell viability using Cell Counting Kit-8 (CCK8) assay. The results exhibited that only oridonin, an ent-kaurene diterpenoid extracted from the Chinese herb *Rabdosia rubescens* [18], selectively inhibits R882H cells within a wide range of concentrations (Figs. 3B and S1F), but not the other 5 compounds (Fig. S1G–K). Furthermore, the half-maximal inhibitory concentration (IC_50_) of oridonin against WT and R882H cells is 6.4 µM and 3.7 µM, respectively, indicating that oridonin is a potential drug candidate targeting *DNMT3A* R882H mutation. Previous reports showed that DOT1L inhibitors compromises DNMT3A-mutant AML [14, 16]. We then evaluated the IC_50_ of two DOT1L inhibitors, SGC0946 and EPZ5676, against R882H cells. The results showed that the IC_50_ of SGC0946 is 22.89 µM, and the IC_50_ of EPZ5676 is 104.3 µM (Fig. 3C). Further analysis revealed that oridonin is ~5-fold and 20-fold more potent in killing R882H cells than SGC0946 and EPZ5676, respectively (Fig. 3C), suggesting that oridonin may be a promising inhibitor targeting *DNMT3A* mutations.

It has been reported that oridonin induces apoptosis in various cancer cells [21–23]. To investigate whether oridonin induces apoptosis in R882H cells, WT and R882H cells were treated with 6 µM oridonin, and the frequency of apoptotic cells was evaluated by Annexin V /DAPI double staining (Fig. 3D). The results showed that the oridonin induces apoptosis in both WT and R882H cells, while the apoptotic rate in R882H cells is twice that in WT cells (Fig. 3E), indicating that R882H cells are more sensitive to oridonin-induced apoptosis. In addition, we found that Dnmt3a R878H (mouse homolog to human DNMT3A R882H) HSCs compromise RIPK1-RIPK3-MLKL-mediated necroptosis [24]. To explore whether oridonin-induced cell death in R882H cells by activating apoptosis alone or both apoptosis and necroptosis, WT and R882H cells were pre-treated with a necroptosis inhibitor RIPA56 (RIP1 inhibitor) [25] or an apoptosis inhibitor z-VAD (pan-caspase inhibitor) for 6 h before oridonin treatment. The flow cytometry results displayed that the cell death of both WT and R882H cells induced by oridonin is significantly blocked by z-VAD but not RIPA56, indicating that oridonin induces apoptosis but not necroptosis in WT and R882H K562 cells (Fig. 3F, G). It has been reported that the expression of RIPK3 is absent in K562 cells which renders them unable to undergo necroptosis [26, 27]. However, the protein level of RIPK3 can be detected in both WT and R882H cells in our situation (Fig. 3H). To further explore the reason why necroptosis is absent in K562, we then sought out to determine the protein level of MLKL in K562 cells and the result showed that MLKL is not expressed in K562 cells (Fig. S1L), suggesting that the defect of necroptosis in K562 cells may be due to the absence of MLKL instead of RIPK3. In line with our previous finding that RIPK1-RIPK3-MLKL-mediated necroptosis is compromised in the HSPCs of R878H mice [24], the protein levels of RIPK1 and RIPK3 are also lower in R882H cells than that in WT cells (Fig. 3H). It is well-known that RIPK1 is a key upstream regulator that controls the activation of apoptosis and necroptosis [28, 29]. When necroptosis is inhibited, RIPK1 can bind with FADD which in turn facilitates activation of caspase-8 independent of the kinase activity of RIPK1, and then caspase-8 executes apoptosis through the cleavage of downstream caspase-3 (Geng et al. [30]). To determine whether oridonin-induced cell death in R882H cells is triggered by RIPK1-Caspase-8-mediated apoptosis, we examined the proteins involved in this pathway by western blot. The results showed that RIPK1 and caspase-8 are upregulated in both oridonin-treated WT and R882H cells, while caspase-3 and cleaved caspase-3 are only increased markedly in oridonin-treated R882H cells (Fig. 3H). This result is consistent with the fact that R882H cells are sensitive to oridonin-induced cell death and apoptosis compared with WT cells (Fig. 3B, E), indicating that oridonin may mainly induce RIPK1-Caspase-8-Caspase-3-mediated apoptosis in R882H K562 cells.

### Oridonin induces the cell death and differentiation of DNMT3A R882 mutant AML cells by activating both apoptosis and necroptosis

**Fig. 4:**
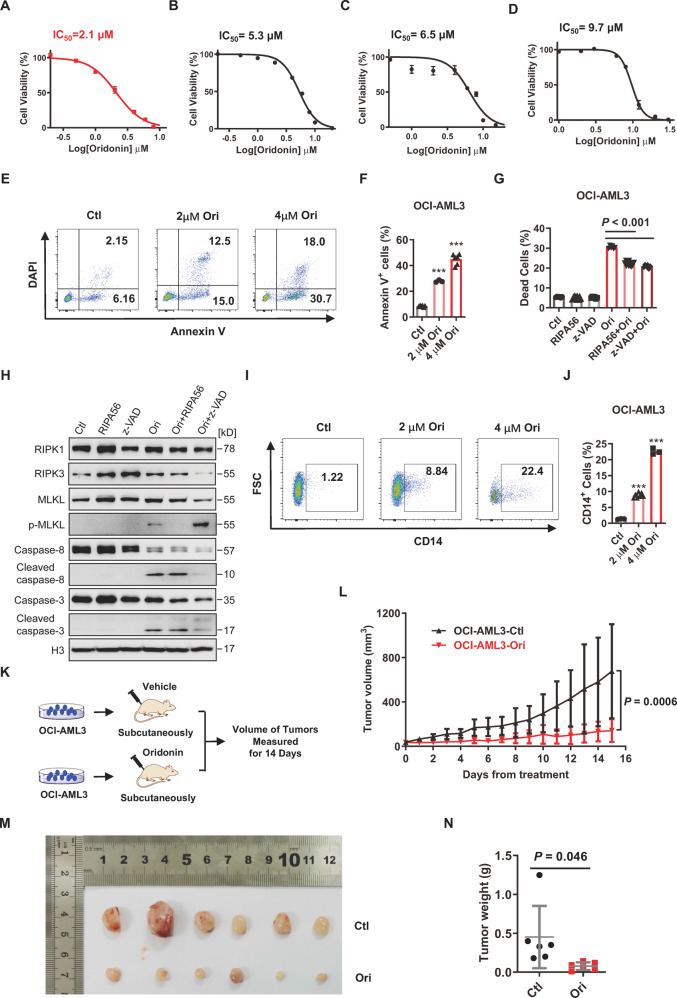
Oridonin induces the cell death and differentiation of DNMT3A R882 mutant AML cells by activating both apoptosis and necroptosis. **A**–**D** The inhibitory effect of oridonin against OCI-AML3 cells (**A**), HEL cells (**B**), KU812 cells (**C**), and HL60 cells (**D**) was evaluated using CCK-8 assay. The IC_50_ of oridonin against the indicated cell lines is displayed in the line plots. Data are represented as mean ± SD, *n* = 3 per concentration from two biological replicates. **E**, **F** Ori induces apoptosis in OCI-AML3 cells. In all, 1 × 10^4^ OCI-AML3 cells were incubated with 2 µM and 4 µM Ori or an equal volume of vehicle. In total, 48 h later, cells were subjected to flow cytometric analysis after staining with Annexin V/DAPI. **E** Representative dot plots of OCI-AML3 cells for apoptotic analysis 48 h after the indicated treatment. **F** The histogram shows the percentage of apoptotic cells (Annexin V^+^) in OCI-AML3 cells with the indicated treatment. All data are represented as mean ± SD and compared to the control group; ***P < 0.001. **G** OCI-AML3 cells (1 × 10^4^ cells per well) were treated with necroptosis inhibitor (RIPA56, 20 µM), apoptotic inhibitor (z-VAD, 20 µM), Ori (2 µM), RIPA56, and Ori or z-VAD and Ori for 48 h. Then, dead cells (DAPI^+^) were analyzed by FACS after DAPI staining. The histogram displays the frequency of dead cells in OCI-AML3 cells with the indicated treatment. Data are shown as mean ± SD from two independent experiments. **H** Immunoblot analysis of the proteins involved in necroptosis and apoptosis pathway in OCI-AML3 cells after treatment with 4 µM Ori for 48 h. **I**, **J** OCI-AML3 cells were treated with 2 µM and 4 µM Ori or an equal volume of DMSO. In all, 48 h later, the expression of CD14 in the surface of OCI-AML3 cells was evaluated by FACS. **I** Representative flow plots of OCI-AML3 cells with the indicated treatment. FSC forward scatter. **J** The histogram exhibits the percentage of CD14^+^ cells in each group. **K**–**N** Oridonin against OCI-AML3 cells was evaluated in a mouse xenograft model. OCI-AML3 cells were subcutaneously inoculated into the right flank of nude mice to generate a mouse xenograft model. In all, 20 mg/kg oridonin or an equal volume of the vehicle were intraperitoneal injections into the left flank of tumor inoculated mice when tumors were measurable. **K** Experimental design. **L** The line plot displays the volume of tumors recorded in each group at the indicated time points during treatment. (*n* = 6 mice per cohort from two independent experiments). Photograph (**M**) and weight (**N** of the indicated tumors removed from mice 15 days after initiation of treatment.

To further explore the inhibitory effect of oridonin against leukemic cells with *DNMT3A* R882 mutation and other clonal-hematopoiesis-caused mutations, we treated OCI-AML3 cells (with *DNMT3A* R882C mutation) [31], HEL cells (carrying *JAK2* V617F mutation) [32], KU812 cells (carrying *ASXL1* R693* mutation) [33], and HL60 cells (p53 null) [34] with oridonin over a series of concentration range (Fig. S2A). The results displayed that the IC_50_ of oridonin against OCI-AML3, HEL, KU812, and HL60 cells is 2.1 µM, 5.3 µM, 6.5 µM, and 9.7 µM, respectively (Fig. 4A–D). Furthermore, the inhibition effect of oridonin against OCI-AML3 cells is approximately 10-fold and 37-fold higher than SGC0946 and EPZ5676, respectively (Fig. S2B–C). These data suggest that oridonin exhibits a selective inhibitory effect on *DNMT3A* R882 mutant cells, and the OCI-AML3 cell line is a good model to study leukemia with *DNMT3A* R882 mutation as described by several studies [5, 10, 14].

In line with the result we observed in K562 cells, oridonin induces OCI-AML3 cell death in a dose-dependent manner. The percentage of Annexin V positive cells increases from 8.51% ± 0.59% in control group to 27.8% ± 0.89% and 45.0% ± 4.13% after 2 μM and 4 μM oridonin treatment, respectively (Fig. 4E, F). To investigate whether oridonin-induced cell death in OCI-AML3 cells by modulating apoptosis or necroptosis, OCI-AML3 cells were pre-treated with RIPA56 or z-VAD for 6 h before oridonin (2 μM) treatment. The results showed that the cell death of OCI-AML3 cells induced by oridonin is partially blocked by both RIPA56 and z-VAD, indicating that oridonin may induce both apoptosis and necroptosis in OCI-AML3 cells (Fig. 4G). To further confirm this result, the proteins involved in both necroptosis and apoptosis pathways were examined by western blot. The result exhibited that the protein levels of RIPK3 and MLKL slightly increase in OCI-AML3 cells upon oridonin treatment, but phosphorylated MLKL (p-MLKL), the final executioner of necroptosis, is induced in oridonin-treated OCI-AML3 cells. In addition, this effect can be blocked by RIPA56 when necroptosis is inhibited or be enhanced by z-VAD when apoptosis is blocked, indicating that oridonin induces cell death in OCI-AML3 cells by modulating necroptosis (Fig. 4H). Furthermore, the protein level of pro-caspase-8 and pro-caspase-3 decreases in OCI-AML3 cells when they are treated with oridonin alone or combined with RIPA56, while the level of cleaved caspase-8 and cleaved caspase-3 increases significantly, and this effect can be blocked by z-VAD, suggesting that oridonin induces apoptosis in OCI-AML3 cells (Fig. 4H). Combining the data above, oridonin may induces cell death of *DNMT3A* R882 mutant cells by activating both RIPK1-Caspase-8-Caspase-3-mediated apoptosis and RIPK1-RIPK3-MLKL-mediated necroptosis. Additionally, the expression of CD14, a mature monocyte marker, increases markedly in oridonin-treated OCI-AML3 cells compared with the vehicle-treated controls in a dose-dependent manner, which provides evidence that oridonin induces differentiation of OCI-AML3 cells (Fig. 4I, J).

Then, the inhibitory effect of oridonin on *DNMT3A* R882 mutant AML cells was also evaluated in vivo. A xenograft model was established by subcutaneous inoculation of 4 × 10^7^ OCI-AML3 cells into the right flank of nude mice. When the recipients developed a measurable tumor at day 7, half of the mice were treated intraperitoneally with oridonin (20 mg/kg) and the rest were administrated with an equal volume of vehicle. The volume of tumors was measured every day by using the published method [23] for 14 consecutive days (Fig. 4K) and the body weights of all recipients were recorded throughout the study. The dose of oridonin was based on a previous report [35]. The results showed that the volume and weight of the OCI-AML3 tumors are significantly reduced after treated with oridonin compared to the control group (Fig. 4L–N). Notably, oridonin treatment does not affect the body weight of mice, indicating no obvious toxicity under this treatment paradigm (Fig. S2D). These data suggest oridonin inhibits leukemic cells with *DNMT3A* R882 mutation in vivo.

### Oridonin inhibits the clonal expansion of Dnmt3a R878H HSCs in vivo

**Fig. 5:**
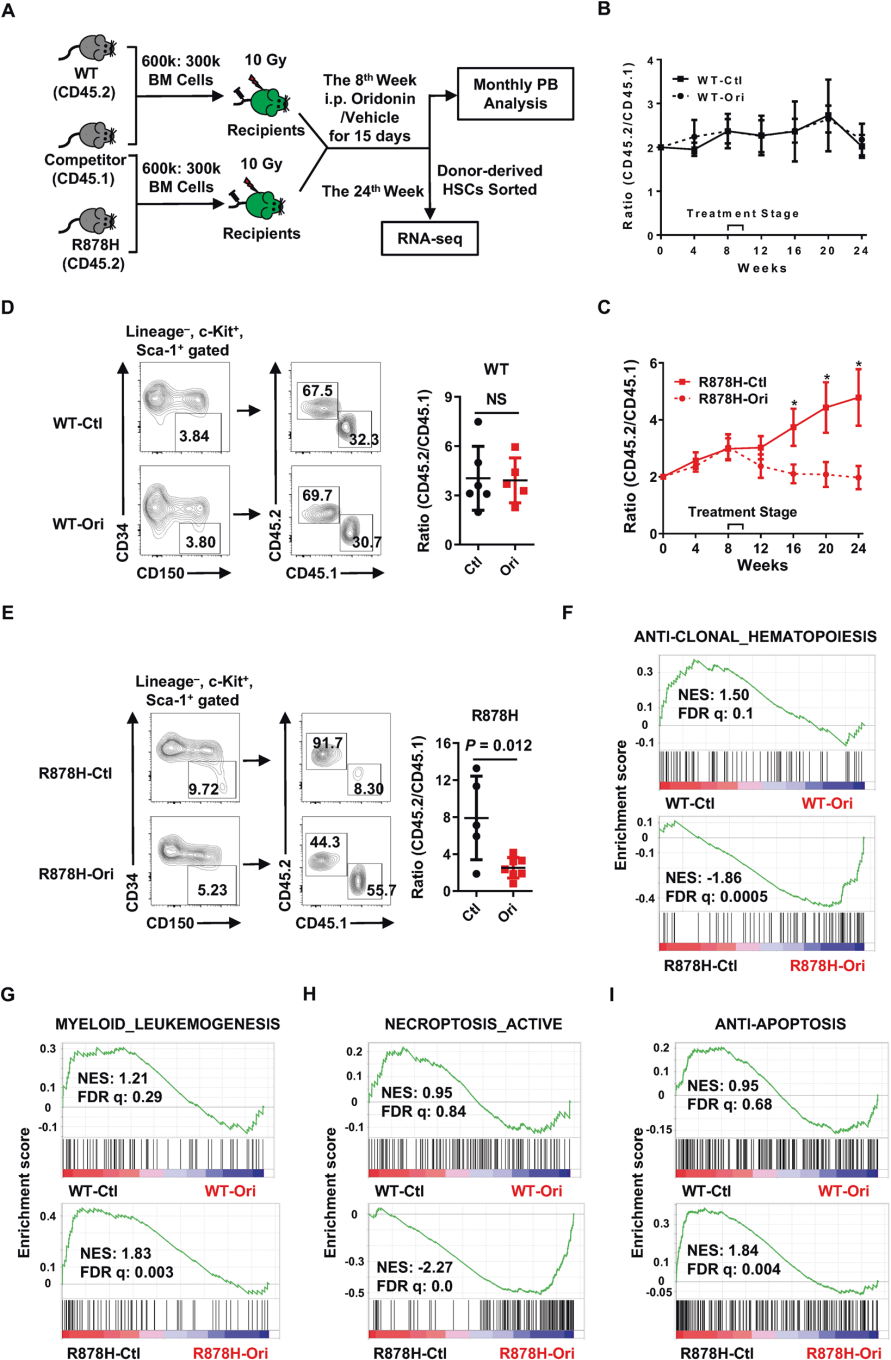
Oridonin inhibits the clonal expansion of Dnmt3a R878H HSCs in vivo. **A**–**E** The inhibitory effect of oridonin against *Dnmt3a* R878H cells was evaluated in vivo. In detail, WT or R878H BM cells (CD45.2) along with competitor BM cells (CD45.1) at a ratio of 2:1 (6 × 10^5^: 3 × 10^5^) were transplanted into lethally irradiated recipient mice (CD45.1/2). Eight weeks post-transplant, the chimeric mice were treated with oridonin (10 mg/kg intraperitoneal injection every day for 15 days) or an equal volume of vehicle (2% DMSO + 20% PEG300 + 78% PBS) accordingly. Chimerism in the PB was analyzed every 4 weeks, and donor-derived HSCs (Lineage^−^ Sca-1^+^ c-kit^+^ CD34^−^ CD150^+^) were evaluated and sorted for RNA sequencing 24 weeks after transplantation. **A** Experimental design. **B**, **C** These line plots show the ratio of CD45.2^+^ cells to CD45.1^+^ cells in the PB of WT ± oridonin (**B**) or R878H ± oridonin (**C**) recipients at the indicated time point. All data above are represented as mean ± SD from two independent experiments; *P < 0.05. **E**, **F** Representative plots from flow cytometry (left) and the histograms (right) show the ratio of CD45.2^+^ HSCs (Lineage^−^ Sca-1^+^ c-Kit^+^ CD34^−^ CD150^+^) to CD45.1^+^ HSCs in WT ± oridonin (**D**) or R878H ± oridonin (**E**) recipients (*n* = 5–7 mice/group from three independent experiments). All data above are shown as mean ± SD. “NS” represents no significance. **F**–**I** These figures display the GSEA of anti-clonal hematopoiesis genes (**F**), myeloid leukemogenesis genes (**G**), necroptosis activation-related genes (**H**), and anti-apoptosis genes (**I**) in WT-Ctl (WT control group) versus WT-Ori (WT oridonin group), or R878H-Ctl (R878H control group) versus R878H-Ori (R878H oridonin group). NES normalized enrichment score, FDR *q* false discovery rate-adjusted *q* values. |NES| > 0.3 and FDR *q* < 0.05 represent a significant difference.

Given that *DNMT3A* R882H promotes clonal hematopoiesis [2, 3], oridonin might mitigate DNMT3A R882H-based clonal hematopoiesis. To test this hypothesis, we transplanted 6 × 10^5^ total bone marrow cells either from WT or *Dnmt3a* R878H mice (a mouse model mimics DNMT3A R882H in humans which was elaborated in our previous work [24] into lethally irradiated recipients together with 3×10^5^ competitor cells. Two months after transplantation, the recipients were treated with either vehicle or oridonin (10 mg/kg) by i.p. injection for 15 days and the chimera of PB was evaluated every 4 weeks until the 24th week (Fig. 5A). Because the recipient mice may exhibit a decreased tolerance upon drug treatment after irradiation, we halved the dose of oridonin based on the xenograft model. The result showed that oridonin exhibits no effect on the reconstitution efficiency of WT bone marrow cells (Figs. 5B and S3A), while the ratio of overall R878H-derived cells declines significantly from 4.79 ± 2.43 in vehicle-treated control to 1.97 ± 1.00 in the oridonin-treated group (Fig. 5C). Furthermore, the above changes in the oridonin-treated group mainly stem from decreases of B cells and myeloid lineages (Fig. S3B).

Then, donor-derived HSCs were analyzed (Lineage^−^ Sca-1^+^ c-kit^+^ CD34^−^ CD150^+^), and we observed that the ratio of WT donor-derived HSCs remains unchanged (3.41 ± 0.89 vs 3.85 ± 2.11) between oridonin-treated and vehicle-treated groups (Fig. 5D). The R878H-derived HSCs dominate in the vehicle-treated group, but it declines significantly after oridonin treatment (Fig. 5E). These data suggested that oridonin inhibits clonal expansion of Dnmt3a R878H HSCs in vivo.

To understand the underlying molecular basis that oridonin inhibits clonal expansion of Dnmt3a R878H HSCs in vivo, donor-derived HSCs were sorted for RNA sequencing. PCA plots (Fig. S3C) and scatter plots (Fig. S3D–E) showed that only a few genes in WT HSCs were disturbed after oridonin treatment, while numerous genes expressed differentially in oridonin-treated R878H HSCs compared with the vehicle-treated control, indicating that oridonin specifically targets R878H HSCs. We then sought to analyze a gene set containing 74 frequently mutated genes driving clonal hematopoiesis and leukemogenesis which was summarized by a recent study [36]. These genes serve as tumor suppressors under normal conditions, but when they are deleted or mutated, clonal hematopoiesis or leukemia occurs frequently. Thus, we defined this gene set as anti-clonal hematopoiesis genes. Gene set enrichment analysis (GSEA) displayed that anti-clonal hematopoiesis genes were significantly enriched in oridonin-treated R878H HSCs (Fig. 5F), which indicates that oridonin inhibits R878H-driven clonal hematopoiesis. Consistently, gene sets associated with myeloid leukemogenesis are markedly reduced in R878H HSCs after oridonin treatment (Fig. 5G), which further suggests that oridonin inhibits R878H-driven myeloid leukemogenesis.

We found that oridonin induces the cell death of *DNMT3A* R882 mutant cells by activating both apoptosis and necroptosis. Thus, we sought to analyze the genes related to apoptosis and necroptosis in oridonin-treated WT and R878H HSCs. The GSEA results showed that the gene sets associated with necroptosis activation are significantly enriched and the gene sets related to anti-apoptosis are remarkably decreased in oridonin-treated R878H HSCs compared with the vehicle control group, while no effects are observed in WT HSCs (Fig. 5H, I). The above results indicate that oridonin specifically induces necroptosis and apoptosis in R878H HSCs, which may explain the reason why oridonin specifically inhibits R878H-driven clonal hematopoiesis.

The above data suggest that oridonin is a potential and promising drug candidate or lead compound for treating DNMT3A mutant clonal hematopoiesis and leukemia.

## Discussion

In this study, we screened drug libraries containing a total of 3917 compounds and identified that oridonin inhibits DNMT3A R882 mutant leukemic cells and Dnmt3a R878H HSCs in vitro and in vivo. The broad-spectrum anticancer effects and the underlying mechanisms of oridonin have been studied for decades [18, 37, 38], and the mechanistic studies primarily focused on genes involved in apoptosis-related and autophagy-related signaling pathways [39–42]. In addition to revealing the proapoptotic effect of oridonin, our study provides evidence for the first time that necroptosis participates in the cell death induced by oridonin in hematological malignancies with DNMT3A mutations. In cancers, including acute myeloid leukemia, the apoptotic pathway is frequently inhibited by upregulating antiapoptotic proteins or downregulating caspase-8 and FADD [43–45]. Thus, necroptosis activated by oridonin can be served as an alternative form of cell death, which may enable oridonin to overcome drug resistance in cancer cells caused by apoptotic inhibition under caspase-compromised conditions. Therefore, necroptosis may play an important role in DNMT3A-mutant leukemia and serve as a potential therapeutic target.

It seems to be contradictory that oridonin up-regulates the expression of caspase-8 in K562 cells, but significantly down-regulates the level of caspase-8 in OCI-AML3 cells. It is reported that caspase-8 is an initiator of the extrinsic apoptosis [46], and necroptosis induction in vitro frequently requires caspase-8 inhibition by z-VAD because caspase-8 cleaves RIPK1 to disintegrate the necrosome [47]. In this study, we found that oridonin induces the cell death of OCI-AML3 cells by activating necroptosis and apoptosis, but necroptosis is absent in K562 cells due to the deficiency of MLKL. Therefore, it is reasonable that the upregulation of caspase-8 in oridonin-treated K562 cells is to activate apoptosis. However, the down-regulation of caspase-8 in oridonin-treated OCI-AML3 cells may facilitate the activation of necroptosis in these cells.

It is reasonable that the expression of the necroptosis-related protein (such as RIPK1, RIPK3, and MLKL) should be upregulated when cells undergo necroptosis by treating with z-VAD and oridonin. However, the level of necroptosis-related proteins does not increase in OCI-AML3 cells undergoing necroptosis, but even shows a downward trend. Previous studies reported that the levels of RIPK1, RIPK3, MLKL, and phosphorylated MLKL in the insoluble fraction increase significantly when cells undergo necroptosis [48]. The insoluble fraction can dissolve in NP-40 buffer with 6 M urea, but not in NP-40 buffer alone [49]. Our protein samples dissolved in NP-40 buffer without urea, and this is the reason why the level of RIPK1, RIPK3, and MLKL seems to be downregulated in cells treated with z-VAD and Ori. However, this result does not affect our conclusion that necroptosis occurs in OCI-AML3 cells, because the phosphorylated MLKL, the final executor of necroptosis, can be detected in these cells.

It is well-known that necroptosis is a “dirty” death, resulting in plasma membrane rupture and the release of damage-associated molecular patterns contributing to the production of pro-inflammatory cytokines [50], and we found that aging-elevated inflammation promotes Dnmt3a R878H-driven clonal hematopoiesis [24] which may conflict with the fact that oridonin inhibits the clonal expansion of Dnmt3a R878H HSCs by activating necroptosis. However, we noticed that oridonin is an ent-kaurene diterpenoid extracted from the Chinese herb *Rabdosia rubescens* which has been used for anti-inflammatory diseases for thousands of years [18, 51]. Indeed, the gene sets associated with inflammation activation are significantly reduced in R878H HSCs after oridonin treatment (Fig. S3F), indicating that oridonin may inhibit the inflammation caused by necroptosis activation.

Oridonin may also be served as a novel agent by combining regular therapies with different or possibly complementary mechanisms of action to improve the efficacy and safety of traditional approaches. A recent study suggests that cisplatin-mediated autophagy is suppressed by oridonin to elevate the sensitivity of ovarian carcinoma cells [52]. Furthermore, oridonin can synergistically enhance the cytotoxicity of doxorubicin and 5-fluorouracil against breast cancer cells and renal carcinoma cells, respectively [53, 54]. In AML patients, carriers of DNMT3A R882 mutation have poor outcomes when treated with anthracycline [5, 6]. It is conceivable that a combination of oridonin with anthracycline may improve the overall survival of AML patients bearing DNMT3A R882 mutation.

Although our data suggest that sensitivity of the leukemic cell lines to oridonin inhibition can attribute to DNMT3A mutation, we are unable to exclude the possible contributions from other genetic or epigenetic alterations. It has been reported that the OCI AML3 cell line also carries an NPM1^c^ mutation in addition to the DNMT3A R882C mutation [55]. However, the K562-R882H cell line developed in this research only differs from the WT control by DNMT3A R882H mutation. R882H cells are inhibited by oridonin with a lower concentration compared with WT cells (Fig. 3B), and we also observed selective inhibition effect of oridonin against Dnmt3a R878H-based clonal hematopoiesis using Dnmt3a R878H mouse model (Fig. 5B, C). Therefore, these observations indicate that the major therapeutic effect of oridonin is attributed to the site specification of the DNMT3A mutation.

The data we obtained from the in vitro and in vivo experiments support the hypothesis that oridonin effectively inhibits DNMT3A R882 mutation-driven clonal hematopoiesis and leukemia, and this provides the preclinical evidence for the possible clinical investigation of oridonin for DNMT3A-mutant leukemia. Furthermore, a prodrug of oridonin called HAO472 has been enrolled into Phase I human clinical trial (CTR20150246; www.chinadrugtrails.org.cn) in China for targeting AML1-ETO [37], which could highly facilitate the translation of our findings into clinical investigations for patients bearing DNMT3A mutations in the near term.

## Materials and methods

### Generation of K562-R882H, K562-tdTomato, and K562-EGFP cells

Three sgRNA oligonucleotides (Table S4) targeting the genomic site encoding *DNMT3A* R882 were annealed and inserted into the pCS vector (Biocytogen, Beijing, China) to acquire Cas9/sgRNA plasmids. An MSD-PCR product (854 bp, primers see Table S4) cloned from *DNMT3A* gene containing the sgRNA target site was cloned into a pUCA(Luc) vector (Biocytogen, Beijing, China) to generate pUCA(Luc)-MSD-DNMT3A plasmid. Then, the pUCA(Luc)-MSD-DNMT3A plasmid was transfected into 293T cells along with three Cas9/sgRNA plasmids, respectively. The cleavage activity of every sgRNA was determined by the Universal CRISPR Activity Assay Kit (Biocytogen, Beijing, China) according to the manufacturer’s instructions. The sgRNA1 was selected for the subsequent gene targeting assay for the strongest cleavage activity. To obtain the donor vector, the 1167-bp fragment DNMT3A-1 and the 1122-bp fragment DNMT3A-2 (primers see Table S4), containing the c.2643C>T (a synonymous mutation) and c.2645G>A (p.R882H) mutations, were ligated into pTV vector (Biocytogen, Beijing, China) containing a loxP-flanked PGK-puromycin resistance (PuroR) cassette. The Cas9/sgRNA plasmids and the donor vector were extracted and purified using the DP117-EndoFree Plasmid Kit (TIANGEN Biotech, Beijing, China). For the electroporation, a total of 10 μg mixed plasmids (5 μg Cas9/sgRNA plasmid and 5 μg donor vector) were added into 100 μL of buffered K562 cell suspension (1 × 10^6^ cells), and this was immediately electroporated at 1400 V for 10 ms with three pulses using the Neon Transfection System (Life Technologies, USA). Transfected cells were then plated into a 96-well plate supplemented with RPMI-1640 medium. Puromycin selection (1 μg/mL) was initiated 3 days after electroporation. Several days later, resistant clones were picked and verified by genomic PCR analysis and Sanger sequencing. Genotyping results suggested that colony 47 was DNMT3A R882H homozygous KI cell lines and hereafter named K562-R882H.

K562-EGFP cells and K562-tdTomato cells were developed from WT K562 cells and K562-R882H cells, respectively. Six sgRNA oligonucleotides (see Table S4) targeting the human *AAVS1* gene were annealed and inserted into the pCS vector (Biocytogen, Beijing, China) to acquire Cas9/sgRNA plasmids. An MSD-PCR product (614 bp, primers see Table S4) cloned from *AAVS1* gene containing the sgRNA target site was cloned into a pUCA(Luc) vector (Biocytogen, Beijing, China) to generate pUCA(Luc)-MSD-AAVS1 plasmid. Then, the pUCA(Luc)-MSD-AAVS1 plasmid was transfected into 293T cells together with six Cas9/sgRNA plasmids, respectively. The cleavage activity of every sgRNA was determined by the Universal CRISPR Activity Assay Kit (Biocytogen, Beijing, China) according to the manufacturer’s instructions. The sgRNA2 was selected for the subsequent gene targeting assay for the strongest cleavage activity. The 773-bp fragment EGFP and the 1484-bp fragment tdTomato were cloned (primers see Table S4) and ligated into a pTg2.0-3XStop vector (Biocytogen, Beijing, China), respectively, to obtain pTg2.0-3XStop-EGFP and pTg2.0-3XStop-tdTomato donor vectors. The Cas9/sgRNA plasmids and the donor vectors were prepared and purified using the DP117-EndoFree Plasmid Kit (TIANGEN Biotech, Beijing, China). For the electroporation, 5 μg Cas9/sgRNA plasmids along with 5 μg pTg2.0-3XStop-EGFP donor vector were added into 100 μL of buffered WT K562 cell suspension (1 × 10^6^ cells), while 5 μg Cas9/sgRNA plasmids together with 5 μg pTg2.0-3XStop-tdTomato donor vector were added into 100 μL of buffered K562-R882H cell suspension (1 × 10^6^ cells). The mixture was immediately electroporated at 1400 V for 10 ms with three pulses using the Neon Transfection System (Life Technologies, USA). Transfected cells were then plated into a 96-well plate supplemented with RPMI-1640 medium. Puromycin selection (1 μg/mL) was initiated 3 days after electroporation. Several days later, resistant clones were picked and verified by genomic PCR analysis and Sanger sequencing.

*DNMT3A* R882 mutations were confirmed in every batch of cells at least once by Sanger sequencing. All cell lines were grown in RPMI-1640 medium supplemented with 10–15% fetal bovine serum and penicillin/streptomycin at 37 °C with 5% CO_2_.

### Cell lines

OCI-AML3 cells were provided by Dr. Min Xiao (an author of this paper) from Huazhong University of Science and Technology. HL60 cells and HEL cells were purchased from DSMZ. K562 cells and KU812 cells were from ATCC. Each cell lines were authenticated by STR profiling and tested for mycoplasma contamination.

### Small molecules screening

All chemicals in 384-well plates were dissolved in DMSO and stored at −20 °C as 10 mM stock solutions until use. In all, 1 × 10^4^ (K562-EGFP: K562-tdTomato = 1:1) cells were seeded into 96-well plates after different chemicals were added into each well (final concentration to 5 μM) by an Echo® 550 Liquid Handler (Labcyte Inc.), and an equal volume of DMSO was used as control. Then, the cell suspension was subjected to a BD LSRFortessa SORP with a BD™ High Throughput Sampler (HTS) for analysis after 48 h. The percentage of GFP^+^ and tdTomato^+^ cells represented the frequency of DNMT3A WT and R882H cells, respectively. Preliminary candidates were identified with a criterion of inhibition ratio no <1.5, and the following formula was used to calculate inhibition ratio: Inhibition ratio = (WT/R882H) _treatment_/(WT/R882H)_control_. Subsequently, preliminary candidates were identified in triplicate to exclude false-positive candidates and obtained six compounds. An FDA-approved library with 2572 chemicals from Selleck and two natural product libraries (Pharmacodia with 936 chemicals and TargetMol with 409 chemicals) were screened. Detailed screening results are shown in Table S1–3.

### Cell viability and proliferation assays

Cell viability of cells treated with small molecules was measured using the Cell Counting Kit-8 (Dojindo, Cat # CK04-11). Generally, K562-EGFP cells, K562-tdTomato cells, or OCI-AML3 cells were seeded in 96-well plates at 1 × 10^5^/ml density with 100 µl medium, and cells were exposed to various concentrations of chemicals in triplicate for 48 h. Then, 10 µl of CCK-8 reagent was added to each well and the resulting cells were incubated at 37 °C for another 4–6 h. The absorbance was measured by an Envision Multilabel Reader (PerkinElmer) spectrophotometry until the maximum absorbance reached the value of ~1 optical density (OD) at 450 nm. Values between the treatment group and vehicle controls were normalized, and measured data were submitted to GraphPad Prism 6 software to calculate the half-maximal inhibitory concentration (IC_50_).

### Western blotting

In all, 1 × 10^6^ cells were lysed with 200 μL NETN buffer (100 mM NaCl, 20 mM Tris-HCl, pH 8.0, 1 mM EDTA, 0.5 mM PMSF, and 0.5% Nonidet P-40) on ice for 30 min. Lysis was completed by sonication and subsequently centrifuged at 14,000×*g* for 5 min. The supernatant was mixed with 2×loading buffer and then boiled for 6 min. Samples were resolved on 10% SDS-PAGE followed by transferring onto a PVDF membrane (BioRad), and then the membrane was blocked by 5% skim milk in TBST buffer before incubating with indicated primary antibodies.

### In vivo tumor xenograft

Four-week-old female BALB/c nude mice bought from Beijing Vital River Laboratory Animal Technology Co., Ltd were subcutaneously inoculated with 4 × 10^7^ OCI-AML3 cells into the right flank. When the tumor was measurable, nude mice were treated with oridonin (20 mg/kg) or an equal volume of vehicle (2% DMSO + 20% PEG300 + 78% PBS) intraperitoneally once a day for 14 consecutive days. Tumor volumes and body weights were measured every day. Length and width of tumor were calipered and the following formula was utilized to calculate tumor volumes: tumor volumes = 4/3 × (width/2)^2^ × (length/2). When tumors reached 2 cm or the mice became moribund, animals were killed and tumors were isolated for analysis. All procedures were performed in the Laboratory Animal Research Center at Tsinghua University and were approved by the Institutional Animal Care and Use Committee of Tsinghua University.

### Competitive bone marrow transplantation

For the oridonin treatment assay, a competitive bone marrow transplantation assay was performed. Briefly, 6 × 10^5^ whole bone marrow cells were freshly isolated from WT or *Dnmt3a* R878H (CD45.2, C57BL/6J mice) and injected intravenously (i.v.) into lethally irradiated (10 Gy) WT recipient mice (CD45.1/2, F1 generated by mating CD45.1 with CD45.2 mice, C57BL/6J mice) together with 3 × 10^5^ WT (CD45.1, C57BL/6J mice) whole bone marrow competitor cells. Peripheral blood from the recipient was analyzed for donor-derived chimerism (myeloid, B, and T cells) monthly. In total, 8 weeks post-transplantation, chimeric mice were injected intraperitoneally with oridonin (10 mg/kg) or an equal volume of vehicle (2% DMSO + 20% PEG300 + 78% PBS) once a day for 15 days, and donor-derived peripheral blood cells were evaluated every four weeks before analyzing donor-derived HSPCs until the 24th weeks.

### Flow cytometric analysis and cell sorting

Non-lysed bone marrow (BM) cells were applied for analysis of HSPC (antibodies containing Lin-APC/Cy7 cocktail, c-Kit APC, Sca-1 PE/Cy7, CD150 PE, CD34 AF700, CD127 BV421, CD16/32 FITC, and CD135 PE-CF594) and lineage (antibodies against Mac1, Gr1, B220, CD3). Chimerism analysis in mature cells from peripheral blood (PB) were lysed by ACK buffer (NH_4_Cl 150 mM, KHCO_3_ 10 mM, Na_2_EDTA 0.1 mM, adjust the pH to 7.2–7.4) and subsequently subjected to flow cytometer after staining with fluorochrome-conjugated antibodies (antibodies containing Mac1, B220, CD3, CD45.1, and CD45.2). A detailed list of antibodies is provided in Table S5.

### RNA library preparation and sequencing

First, 200 donor-derived HSCs (CD45.2^+^ Lineage^−^ Sca-1^+^ c-kit^+^ CD34^−^ CD150^+^) from oridonin-treated recipients were sorted into lysis buffer directly. The transcriptome libraries were prepared using the Smart-seq2 method. Then, Agilent Bioanalyzer 2100 system (Agilent Technologies) was used to assess the insertion size, and HiSeq PE Cluster Kit v4-cBot-HS (Illumina) was used to perform the clustering of the index-coded samples according to the manufacturer’s instructions. Finally, the libraries were sequenced using an Illumina Hiseq platform with a 150-bp paired-end.

### RNA sequencing data analysis

First, software Trim Galore (v0.6.6) was used to perform quality control and adapter trimming of the original cleaned reads of samples. Trimmed reads were mapped to the mouse reference genome GRCm38 from Ensembl database through hisat2 (v2.2.0) [56], and the reads count matrix was acquired with HTSeq (0.12.4) [57]. Then, DESeq2 (an R package) [58] was used to normalize the gene counts and assess the differential expression levels of samples between two different groups. Genes with both FDR-adjusted *p*-value below 0.05 and fold change above 1.5 were considered as differentially expressed genes. GSEA software (http://software.broad-institute.org/gsea/) was applied to perform Gene Set Enrichment Analysis (GSEA) with PreRanked weighted mode [59]. Anti-clonal hematopoiesis-associated gene signature, myeloid leukemogenesis-associated gene signature, necroptosis activation-associated gene signature, and anti-apoptosis-associated gene signature were collected from literature and the gene lists were shown in Table S6. Principle Component Analysis (PCA) was used to reduce the dimension, which was conducted by using the R package labdsv.

All sequencing raw data were deposited into the National Center for Biotechnology Information Gene Expression Omnibus with accession number GSE174362.

### Statistical analysis

All data are shown as mean ± SD. A two-tailed unpaired Student’s *t*-test was used for statistical significance analysis after testing for normal distribution and data were plotted using GraphPad Prism 6 software.

## Supplementary information

Supplemental Figure 1

Supplemental Figure 2

Supplemental Figure 3

Supplementary figure and table legends

Table S1

Table S2

Table S3

Table S4

Table S5

Table S6

## Data Availability

Further information and requests for resources and reagents should be directed to the Lead Contact: Jianwei Wang (jianweiwang@mail.tsinghua.edu.cn). New experimental animal models and cell lines generated in this study are listed in the Table S5 and available upon request.
