# Supplementary figures and images for: Oridonin inhibits DNMT3A R882 mutation-driven clonal hematopoiesis and leukemia by inducing apoptosis and necroptosis
Source: Cell Death Discov. 2021 Oct 18;7:297. doi: 10.1038/s41420-021-00697-5 (PMC8523644; doi:10.1038/s41420-021-00697-5)

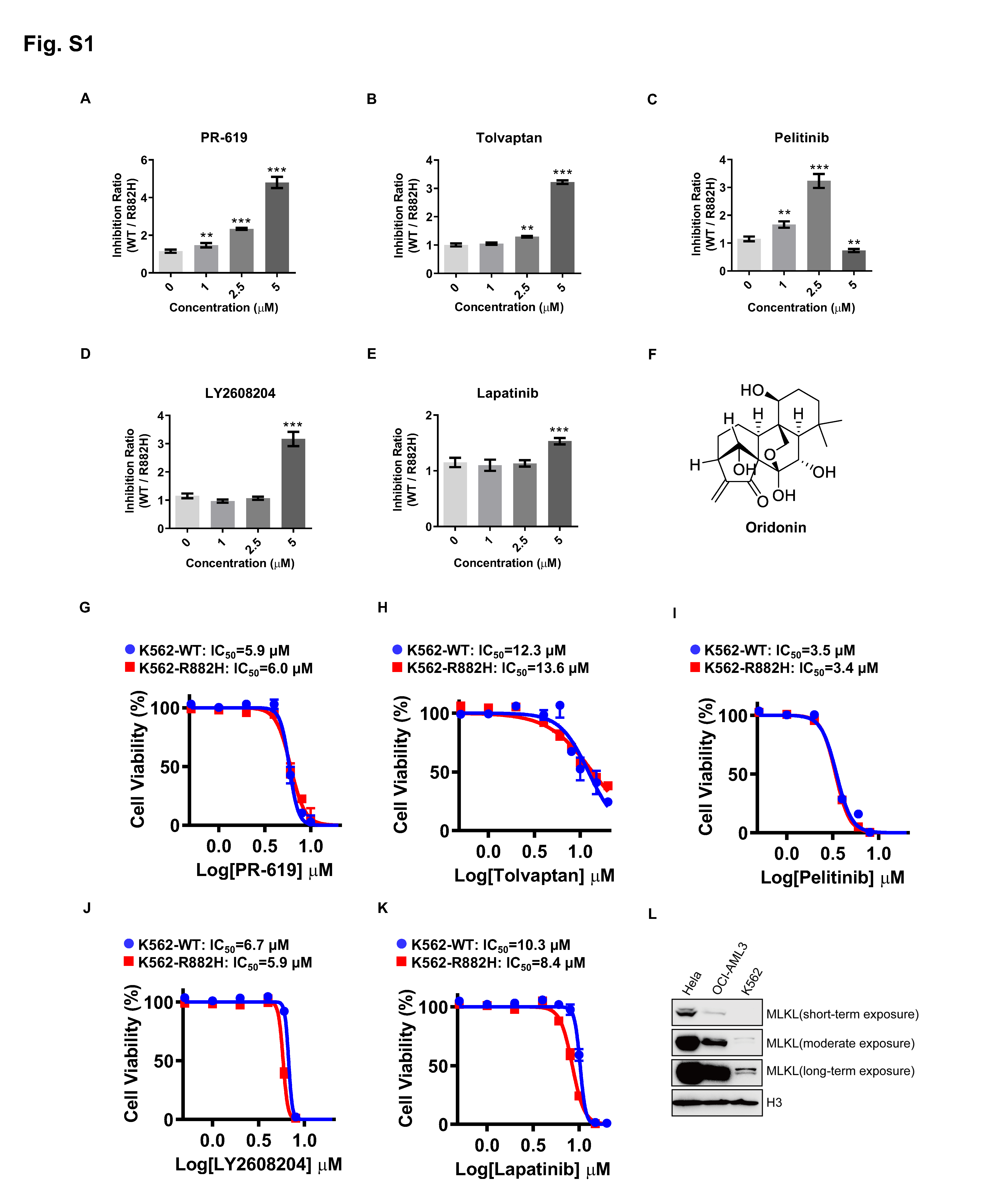

Supplement: Supplementary file 1 — Supplemental Figure 1 [file 41420_2021_697_MOESM1_ESM.tif]

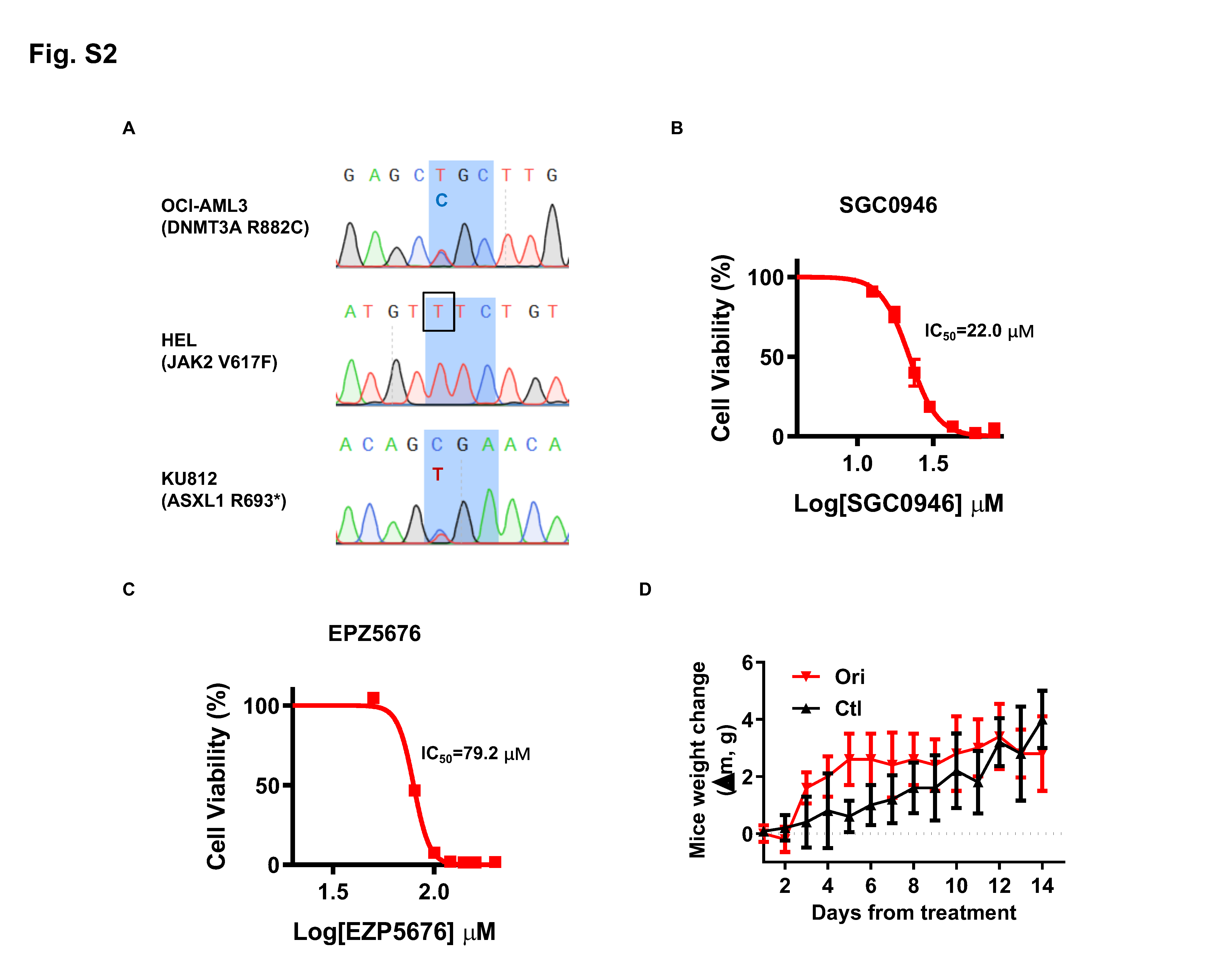

Supplement: Supplementary file 2 — Supplemental Figure 2 [file 41420_2021_697_MOESM2_ESM.tif]

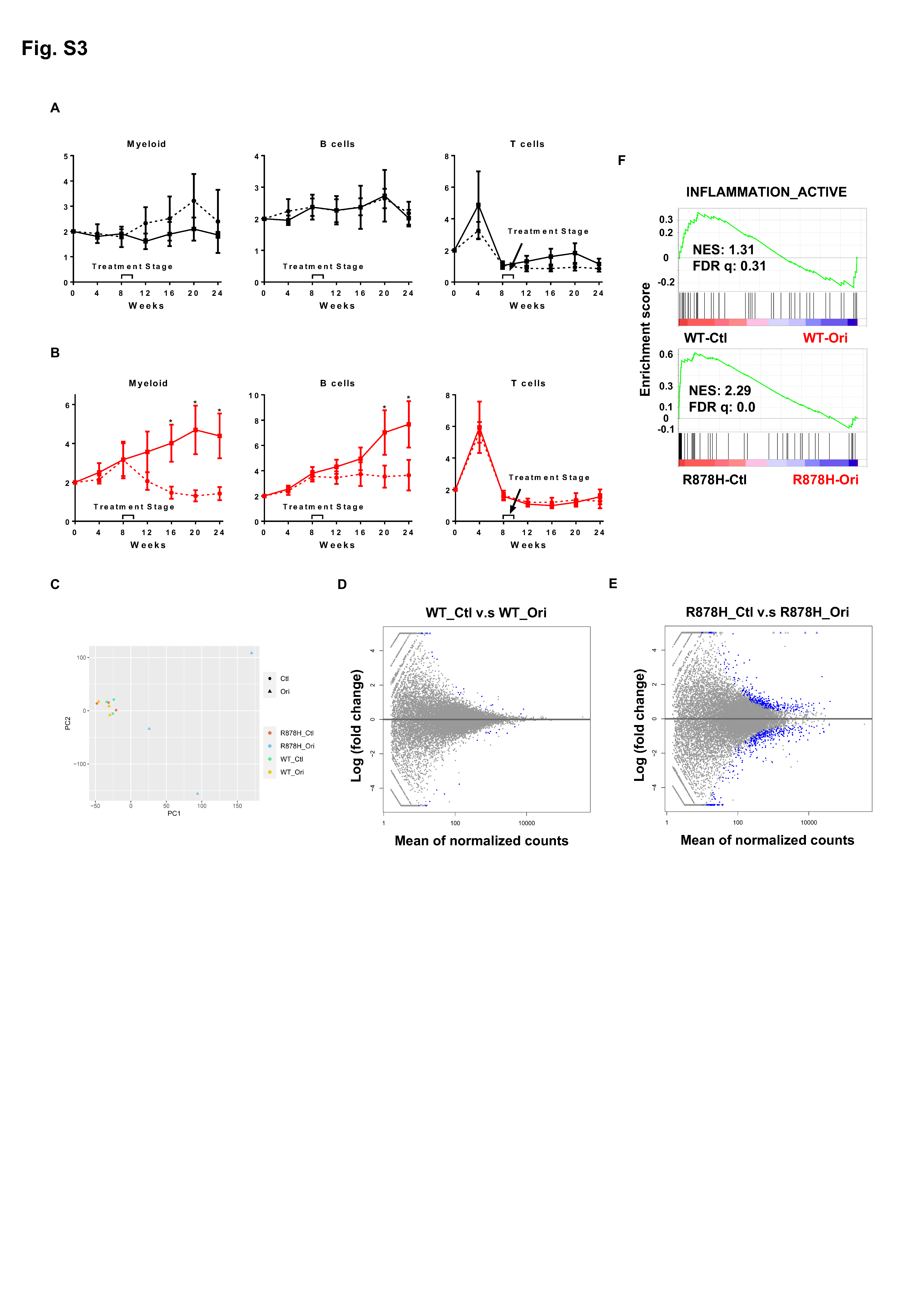

Supplement: Supplementary file 3 — Supplemental Figure 3 [file 41420_2021_697_MOESM3_ESM.tif]
