# Supplementary material for: Oridonin inhibits DNMT3A R882 mutation-driven clonal hematopoiesis and leukemia by inducing apoptosis and necroptosis
Source: Cell Death Discov. 2021 Oct 18;7:297. doi: 10.1038/s41420-021-00697-5 (PMC8523644; doi:10.1038/s41420-021-00697-5)
Supplement: Supplementary file 4 — Supplementary figure and table legends [file 41420_2021_697_MOESM4_ESM.docx]

**Figure S1 Inhibitory effect of candidate small molecules targeting DNMT3A R882H cells (Related to Fig. 3).**

(A-E) The histograms show the inhibitory effect of PR-619 (A), Tolvaptan (B), Pelitinib (C), LY2608204 (D) and Lapatinib (E) against R882H cells at different concentrations. The inhibition ratio shown in the vertical axis was calculated using the percentage of WT cells divided by R882H cells. Briefly, 5×10^3^ K562-EGFP cells along with 5×10^3^ K562-tdTomato cells were seeded into 96-well plates. Then, cells were treated in triplicate with the indicated compounds at different concentrations and all cultures were subjected to FACS analysis 48 hours later. All data above are shown as mean ± SD and compared to the vehicle control; ***P* < 0.01, and ****P* < 0.001.

(F) The illustration shows the chemical structure of oridonin.

(G-K) 5 candidates obtained from HTS were further evaluated using a CCK-8 kit. Cells were seeded into 96-well plate at a density of 1×10^4^ cells per well and cultured with different concentrations of the indicated chemicals for 48 hours. Then, 10 µl of CCK-8 reagent was added into each well and the resulting cells were incubated at 37 °C for another 4 to 6 h. The absorbance was measured by spectrophotometry. The line plots exhibit the inhibitory effect of PR-619 (G), Tolvaptan (H), Pelitinib (I), LY2608204 (J) and Lapatinib (K) against R882H cells at different concentrations. All data above are shown as mean ± SD.

(L) Immunoblot analysis of MLKL in Hela cells, OCI-AML3 cells and K562 cells.

**Figure S2 Oridonin Induces the cell death and differentiation of DNMT3A R882 mutant AML cells by activating both apoptosis and necroptosis (Related to Fig. 4).**

(A) Sequencing analysis of the genomic site encoding *DNMT3A* R882 (the shadow) in OCI-AML3 cells, the genomic site encoding *JAK2* V617 (the shadow) in HEL cells and the genomic site encoding *ASXL1* R693 (the shadow) in KU812 cells (primers for sequencing are detailed in Table S4).

(B-C) The inhibitory effect of SGC0946 (B) and EPZ5676 (C) against OCI-AML3 cells was evaluated using a CCK-8 kit. The IC_50_ of two compounds is displayed in the line plots. Data are represented as mean ± SD, *n* = 3 per concentration from two biological replicates.

(D) The line plot displays the body weight changes of the indicated xenotransplant mice during treatment (*n* = 4-6 animals per cohort).

**Figure S3 Oridonin inhibits the clonal expansion of Dnmt3a R878H HSCs *in vivo* (Related to Fig. 5).**

(A-B) These line plots show the ratio of CD45.2^+^ myeloid, B and T cells to the corresponding CD45.1^+^ cells in the PB of WT ± oridonin (A) or R878H ± oridonin (B) recipients at the indicated time point. All data are shown as mean ± SD from two independent experiments; **P* < 0.05.

(C) The PCA (principal component analysis) plot shows the classification of WT control (WT-Ctl), WT oridonin (WT-Ori), R878H control (R878H-Ctl) and R878H oridonin (R878H-Ori) group based on the normalized RNA-seq levels of all mouse genes.

(D-E) The scatter plots display the overall transcriptome changes in WT-Ctl versus WT-Ori HSCs (D), or R878H-Ctl versus R878H-Ori HSCs (E). Blue dots represent genes with adjusted *P* value below 0.05.

(F) The figure shows the GSEA of inflammation activation-related genes in WT-Ctl versus WT-Ori HSCs, or R878H-Ctl versus R878H-Ori HSCs. NES, normalized enrichment score; FDR q, false discovery rate-adjusted q values. |NES|>0.3 and FDR q<0.05 represent significant difference.

Table S1. FACS Screening Results-Selleck

Table S2. FACS Screening Results-TargetMol

Table S3. FACS Screening Results-Pharmacodia

Table S4. Primers for Genotyping and qRT-PCR

Table S5. Key resources used in this study

Table S6. GSEA gene sets
